# 

**Published:** 1847-10

**Authors:** 


### POSTSCRIPT

TO

### No. XLVIII OF THE BRITISH AND FOREIGN MEDICAL REVIEW.

BY THE EDITOR AND PROPRIETOR.

---

With the present Number, the 'British and Foreign Medical Review,' after an uninterrupted course of quarterly publication for twelve years, comes to a conclusion;—at least, under its original form and designation, and as *my* Journal. On such an occasion, it seems but courteous to my readers, and to the numerous friends who have so warmly supported me, to say something by way of leave-taking; and it may be both interesting and useful to some of them, as well as to others, now or hereafter, if I also give a few particulars respecting the history and fortunes of a work which the profession has been pleased to regard as valuable and influential. Having been the exclusive originator, planner, proprietor, and conductor of the Journal, I possess means of making an exposition of this kind, which have seldom existed in similar cases.*

For many years before the commencement of the Review,—in common I believe, with all who had any pretensions to be judges in such matters, and who had paid even moderate attention to the state of medical literature in this country,—I had been deeply impressed with the deficiencies of the medical press generally, more especially in the department of criticism, as exhibited in the pages of the more popular journals. In some of these, not only was the general style of the writing of the coarsest and most slovenly character, but, what was still more lamentable, the judgments and opinions passed on the books reviewed were, not merely crude and unsifted, but often most incorrect and unfair. Generally speaking, the articles mainly consisted of extracts, connected by little scraps from the pen of the reviewer, made up of the merest truisms and dullest common-places, usually of a more or less laudatory character. The principle of indiscriminate laudation would, indeed, almost seem a necessary part of such scissors-work and patch-work reviewing; as a critic while passing a severe condemnation on a book, could hardly have the face to treat his readers to voluminous extracts from its pages. This general inferiority of medical criticism and false appreciation of literary merit, and these defective judgments had another and facile source in the unfortunate system of *gratuitous contribution* generally prevalent. An editor whose professed system was not to pay for contributions, was naturally enough ready to receive help in his laborious task, and was not likely to be over-fastidious in questioning the capabilities of his volunteer writers, or in canvassing the motives which induced them to write in particular cases. It may thus easily be understood how articles might find their way into journals so conducted, written by very incompetent or improper persons; by raw, unpractised youths anxious to see their lucubrations in print; by the friends of the authors whose works were

* My excellent friend, Dr. Conolly, co-operated with me as Editor during the publication of the first few numbers; but, although his name remained afterwards on the title-page, he took no share whatever in the editing. The readers of the Review, however, had often the benefit of his admirable talents as a contributor.

reviewed; or even by the authors themselves.* *Foreign* works were, of course, almost virtually excluded from publications of the kind referred to, except occasionally when an easy French volume afforded an opportunity to some youthful literary aspirant of exercising a laudable industry in mastering the heads of a practical treatise, and giving an epitome thereof: criticism in such a case was not to be expected. Other reasons might be given for the low standard of medical criticism at the period referred to, as well as for the very limited scope of the journals exhibiting it; but what I have stated is sufficient to show that at the time when this Journal was set on foot, there was great need for a Review of a superior stamp; and there seemed ample justification, both professionally and commercially, for the attempt to establish the 'British and Foreign Medical Review' on a broader basis and with higher aims.

The plan and objects of the new Journal were long under consideration, and were completely matured previously to the appearance of the first number. They were intended to save it from the various evil influences which were believed to interfere so seriously with the utility and value of some other journals, as well as from evils of a different kind, which, without precaution, might spring up in its own path.

The leading principles on which it was determined to conduct the Journal were—in the first place, a thorough impartiality and independence of criticism, and a determination to speak the truth both as to books and men, without regard to person or place; secondly, the establishing, as far as this was practicable, of a tribunal, competent by the character, learning, and experience of its members, not merely to judge truly of the merits of the different works that came under notice, but to correct their errors and supply their deficiencies; thirdly, the regarding works under review as literary as well as technical productions, and thus holding the authors responsible for the form as well as the matter of their productions—for the language as well as the science; lastly, the considering the medical literature of all countries as equally entitled to notice, that is, reviewing books according to their actual merit and importance, without reference to nation or language.

It was proposed to carry these principles into effect by means of certain well-considered arrangements. As these were adopted from the very first, and adhered to as closely as possible through the whole course of the Journal, there will be little impropriety, while noticing them, to speak of them either in the past tense, or prospectively, as may be most convenient. The following are the more material of these arrangements:

1. No gratuitous articles to be admitted; and the remuneration to be such as to justify the Editor in expecting the co-operation of competent writers.†

2. All books thought deserving of notice, whether British or foreign (including Journals), to be purchased, if not presented to the editor.

3. The different writers to be enjoined the strictest impartiality. As promotive of this, the Editor was careful to avoid intrusting particular books to writers who, from circumstances, might be presumed to be more or less biassed against or in favour of the authors, from being known to have been or to be at variance, or particularly intimate with the author, from the

* I think it right to state that I do not intend these strictures to apply to the 'Edinburgh Medical and Surgical Journal.' Its reviews were, on the whole, good; but its limited plan entirely prevented it from giving anything like a comprehensive account of books.

† The precise remuneration was seven shillings and sixpence per printed page, or six pounds per printed sheet of sixteen pages, extracts being reckoned as original matter.

parties living in the same town, from being rivals in practice, or from being the authors of rival works, &c. &c. On the same grounds it was also an invariable rule never to allow the name of the publisher of any book to appear at the head of an article. Still further aid towards insuring impartiality (as well as other important ends, which need not be specified) was derived from the regulation, seldom neglected, of the books reviewed being returned along with the MS. articles. The Editor had thus the opportunity of again examining the work criticised, if at any time it should appear to him, while reading the articles in manuscript, that there were any doubts of the writer's perfect fairness. The contributors will bear witness that this was a duty which the Editor never shrank from, and rigidly exercised when he thought he had reason on his side.

4. It was felt to be the most important duty of the Editor to endeavour on all occasions to obtain the best articles, by securing, if possible, not merely the best writers, but writers who, from circumstances, were specially qualified as judges on the subjects of the respective works. This end it was impossible always to attain in a perfect manner, but assuredly no pains were spared to attain it. It was on this principle that, although some of the Editor's literary friends were more or less constantly contributing to the Review, there was nothing like a fixed band of contributors, to whom a certain quantity of work was habitually to be intrusted, and who were alike ready and qualified for all sorts of books and all sorts of subjects. In proof of this rule being constantly attended to, it may be mentioned that the lists of the writers in any two consecutive numbers differed greatly, that the average number of contributors to a single number varied from twelve to twenty, while the total number of gentlemen engaged in writing for the Review at one time, say within the period of a year, was forty or fifty, and sometimes more than this.

5. It was an invariable rule with the Editor to read over carefully the MSS. of all the articles,* and it was always an understanding formally come to between him and the contributors, that he should reserve to himself the power of altering articles, by omission, addition, or otherwise, and even to reject them altogether. It is but justice to his literary friends here to state, that there were rarely any difficulties interposed in the way of alterations: the total rejection of an article was a very rare occurrence.

6. Partly from an indisposition to engage in compositions requiring much labour of thought or much continuous study, and partly from want of time, the Editor rarely supplied any elaborate articles from his own pen. By thus saving his time at the expense of his money (as indolent men are wont), he was not only enabled to perform his common editorial duties more effectually, but he was left much more in the condition of an impartial judge of the articles that came under his examination, than he possibly could have been had many of these been his own. He can sincerely and without any affectation add his conviction, that, by this abstinence, he consulted not merely the greater impartiality, but the greater excellence in every way, of the writings in the Journal. This disclaimer respecting contributions from his own pen, the Editor intends to apply to the longer or more elaborate articles only; he certainly wrote many brief notices of books, and added or re-wrote passages of more or less length in very many of the longer articles contributed by others.

* To be scrupulously accurate, I should say that articles written by experienced and careful contributors were occasionally not read until they were in proof; but I think I can safely say that the total number of articles so privileged was under twelve during the twelve years.

7. Partly from the fear of being behind the proper day of publication, but principally to allow ample time for the examination of MSS. previously to going to press, it was a general rule with the Editor in making his arrangements, not to consider whether any particular article was wanted for a particular number, but simply whether such an article was desirable for the Review. If it was deemed desirable for the Review, and was found when written to be a good article, it was sure to find a place, some time or other. Besides the advantages mentioned, adherence to this rule had some other excellent consequences, which tended greatly to the comfort of the Editor and his literary friends. Thanks to it, he was seldom under the necessity of hurrying his contributors, and rarely under the necessity of hurrying either himself or the printer; his MS. boxes were so well stored that he was enabled to give out for particular numbers the articles that seemed to him most suitable; and the printer's proofs coming in, as they did, singly and during every week of the year, were read at leisure and without much inconvenience or trouble.*

8. The fortunate state of universal peace that prevailed, and the unlimited intercourse with foreign nations, left no difficulty in the way of procuring publications from abroad; and it is greatly to the credit of the profession that I readily found, in our own country, men capable of conveying to their professional brethren, not merely a full account of the contents of foreign works, but also a sound estimate of their merits as scientific productions. Every number of the Review will attest the truth of this as regards French and German literature, while most of the volumes bear similar witness in respect of the Italian, Danish, and Swedish, as well as of the Greek and Latin. I believe I may safely add that the Review itself was no inconsiderable cause of the greatly-increased knowledge of foreign languages, now so honorably characteristic of the better-educated members of the profession in this country. Many young men, to my knowledge, were stimulated to study the continental languages by the treasures they found extracted from them in the Review; and some of my literary friends, in their zeal to serve me and the Journal, actually set themselves to master some of the stranger tongues, in order that they might supply analyses of the more valuable works written in them.

The first number of the Review was published on the 1st of January, 1836, and the succeeding numbers appeared on the first day of each subsequent quarter, respectively, without one single exception. Indeed, the precision of the arrangements was such that the Number was almost always ready a week or a fortnight before the day of publication. By this means the Journal was enabled to appear on quarter-day in Edinburgh and Dublin, as well as in London. At the suggestion of the publisher, no less than 2250 copies were printed of the first number, 1000 copies being intended for distribution to societies, clubs, editors, &c.; 2000 were printed of the three following numbers; and 1750 of the numbers of the second year: by this time the probable standing sale of the work having been ascertained, the number was, in 1838, reduced to 1500, and has not varied since.

The Review was considered by "the trade," at the close of the first

* During the twelve years the press never stopped—the new number being generally begun to be *printed* before the previous one was *published*. It would be unjust to my excellent printers, Messrs. Adlard, and their intelligent manager, Mr. Tucker, if I did not here express my thanks to them for the uniform readiness and attention they showed in meeting all my wishes respecting the printing of the Review, as well as for their excellent and accurate typography.

year, to be eminently successful, as it very speedily reached an amount of steady sale which had heretofore been regarded as very satisfactory for a periodical work devoted to medical science. It was so considered by myself; and I should be very ungrateful to my numerous and kind friends if I did not take this opportunity of thanking them most cordially for the liberal support extended to the Review during its whole course, but more especially at its commencement. All things considered, the patronage it received was quite as general and extensive as could reasonably be expected. Assuredly, I cannot but regret that the taste for the higher departments of medical science has not been and is not more general in this country; and I take my share of shame, as a member of the profession, that our better literature is not more substantially patronised by those who can afford this patronage; but I am far too sensible of my own deficiencies in this respect, to be in a position to blame others for not buying books—however good—on principle and for the pure love of literature and science. When the taste and the love for such things shall grow stronger and spread wider—and I believe they are now doing so—the gratification of the desire thence arising will follow as a matter of course, and then we shall have patronage of authors and books and journals even to the contentment of the commercial heart itself. In the meanwhile we must not quarrel with what is equally in the natural course of things.

In the table given below, the actual sale of the Review is specified for each year, from the commencement to the close of the work, together with the cost of production, and the total money-produce. From this table it will appear that the Review, regarded as a commercial speculation, was by no means a successful undertaking; as it left the Proprietor very considerably a loser, even while making a present to the concern (as he has done in drawing up the table), not only of the interest of the money expended in the first instance, but of the whole of his editorial labour, and all his own literary contributions. Had I added these latter items to the other items of expenditure, as properly speaking I ought to have done, the balance, on the wrong side, would have been not a little increased. At the very lowest rate of payment that could be thought of for such services, say £50 per number, or £200 per annum, the total balance against the work would have been increased by £2400, and thus raised to £2945 10*s*. instead of £545 10*s*., as it there stands. It is, however, but fair to deduct from the adverse balance the sum of £500 received by the Proprietor for the copyright of the Review. The stock of books remaining on hand is also worth something; but the amount reckoned as likely to accrue from this will be required to cover the loss expected to be incurred by the publication of the General Index now in hand, and which has always been regarded as an essential portion of the work.

Although in originating the 'British and Foreign Medical Review', I certainly calculated on its eventual success as a commercial undertaking, feeling confident, on general principles, that the superiority of the article intended to be produced, would, in the long run, ensure a decidedly remunerative sale,* I had so many other strong motives for establishing it, and for carrying it on, that the discovery, a few years after

* Some of my original schemes respecting the Review were very grand. Had its commercial success justified the outlay, it was one of my plans to have a German translation of it made in Hamburgh, for circulation among the German states, and also a reprint of it in America. This last has been done lately by an American publisher at his own cost.

its first publication, that it entailed a loss instead of a gain, interfered in no degree with my determination to persevere in the undertaking, or with the zeal or satisfaction—I might indeed say the delight—with which I continued my labours. It was only on attaining that great milestone of man's life, THREESCORE, that I began to think it would be as well to relinquish a pursuit which had for twelve years absorbed much of my time and my best faculties, and to look round me and see whether some "pastures new" could not be found which might advantageously take the place of the old. I felt the remonstrance of Lucretius's NATURA to be almost as applicable to myself as to his Man in general on a more solemn occasion:

> —— "Si grata fuit tibi vita anteacta priorque
> Et non omnia pertusum congesta quasi in vas
> Commoda perfluere atque ingrata interiere,
> Cur non ut plenus vitæ conviva recedis ?"

And when I felt, in addition, that the task I was engaged in was in no respect compulsory, and that all considerations of a pecuniary kind were decidedly against its continuance, my mind was soon made up as to the expediency of a change. Accordingly, the discontinuance of the Journal was no sooner thought of than decided on. Up to the time in which this decision was come to (in January last), I can honestly say that my exertions in the cause of the Journal, such as they were, had undergone no diminution, and that I had lost none of the feelings of satisfaction which usually accompanied them; and it is with real comfort that I am able to add, in now closing my editorial labours, that the same feelings have continued to attend me up to this, the last scene of the little history which I am now, rather too egotistically I fear, obtruding on my reader's notice.

But although commercially a failure, in a literary and scientific point of view the 'British and Foreign Medical Review' must be regarded as a very successful publication. Its home circulation, compared with that of other quarterly and monthly medical publications, was large, and it had a much greater circulation abroad than any of them. It failed as a commercial undertaking, not so much because the extent of its sale was actually small for a medical publication, but because its cost of production, so much beyond that of any other medical journal, was disproportioned to this sale. In every other respect, *except this of sale*, the success of the Review was great, and entirely fulfilled every sober hope and expectation entertained at its establishment.

And here, while speaking of the literary and scientific merits of the Review, I beg to remind the reader of a statement made above as to the very small share I myself had in the actual composition of its contents: so small, indeed, was my share, that I consider myself entitled to pass a judgment on the merits of the whole work, with almost the same freedom as if I had had no connexion with it. And the delicacy I might feel on this head is yet further removed by the two following considerations—first, that I should not otherwise be able to do justice to the excellent friends who did write the Journal; and, secondly, that opinions of fully as favorable a character as those I entertain, have been expressed to me, hundreds of times, in speech and by letter, by most competent judges in this and other countries. I therefore do not hesitate to repeat that the success of the Review was in every respect great—I might indeed say triumphant—except as a mere money-making speculation. It succeeded in establishing, for the first time, in medical literature, a high critical tribunal analogous to those which had previously conferred so great benefits on general literature. It investigated the literary as well as the scientific merits of books, and boldly pronounced its opinions of them, whether favorable or unfavorable, without regard to the name or station of the authors. It made the medical profession of this country as familiar with the productions of foreign countries as with their own. It allowed no improvement, or discovery, or new fact of interest or curiosity to be promulgated in any part of the world, without conveying it speedily to its readers. In giving its judgments, it was altogether beyond the influence of authors or publishers, or of any individuals whatever; and if its judgments were not always right, they were never prompted by unworthy motives. A few respectable individuals intolerant of fair criticism from overweening vanity or self-conceit, several weak men whom nature never intended to meddle with the pen, and sundry intra-professional quacks of various rank and complexion, have occasionally attempted to raise the cry of partiality and injustice against its decisions; but I here boldly appeal to all disinterested men and good judges, whether, taken as a whole, the opinions promulgated, and the critical awards made by the writers in the Review, from its beginning to its termination, have not been remarkable for their honesty, accuracy, and justice. That they were *always right,* no man of common sense could for a moment pretend; but that they were as nearly right as the means at the Editor's command could make them, is confidently asserted. As consequences of this character, it is still further maintained—*firstly* (in reference to the past), that the Review has of late years been regarded by the best-informed members of the profession, in this and other countries, as the highest authority on medical subjects; and, *secondly* (in reference to the future), that, by showing what a learned, independent, and liberal review is and can effect, it has not only prevented, in time to come, works of inferior character and merit from finding acceptance with the profession in this country, but has assured the perpetuity of a work or works of the same high stamp as itself.

After thus distinctly and deliberately committing myself to maintain for the Review a character of such comparative excellence, my readers may naturally expect from me some explanation of the fact that the circulation of the work did not become more general. It appears from the table, given below, that the highest quarterly sales (including *back* numbers) were for the years 1840 and 1841, amounting, on the average, to 1415 and 1410 respectively; but the sale of the current numbers—in other words, *the circulation* of the Review—was considerably below this, never reaching to 1400. Premising that the amount of *sale,* and the amount of *circulation* of a journal, are not always or generally the same thing, I think a good many reasons may be given, some weaker, some stronger, why it happened, that the sale of the 'British and Foreign Medical Review' was not greater.

1. Since the introduction of the lighter medical publications—the weekly journals—the larger periodical publications have generally declined in circulation, and perhaps the quarterly, as more expensive, more than the others. Many persons are naturally tempted by the small cost of the weekly journals, by the attraction of a quickly-recurring publication, by the facility of conveyance by post, by the gossip and news, &c. constituting an essential character of such publications, to become subscribers to one or other of them; and either from economy, or other motives, cease to take in any other journal. *Retrospects* and *Abstracts,* lately published in a compendious form half-yearly, have still farther encroached on the circulation of the larger journals.

2. The establishment of medical book clubs or reading societies throughout the kingdom, decidedly more numerous of late years, has very considerably affected the sale of the larger journals. In these clubs, established in almost every town, generally speaking only a single copy of a journal is usually taken, and this is made to supply the whole society, including, it may be, ten, twenty, or more members. No doubt these clubs create a sure demand for a very considerable number of copies of the various journals published; but as in most of them *all* the journals are generally ordered, as a matter of course, no room is left for the operation of special preference founded on the individual merit of the publications. The number of persons thus enabled to *see* the medical journals is no doubt large; and the casual, temporary, or late sight of them thus afforded, is sufficient to satisfy the curiosity of a majority of readers.

3. Zealous young literary men, especially young editors or projectors of Journals, entertain a very exaggerated opinion of the actual number of readers of medical journals or other medical books in this country. Feeling how interested they themselves are about improvements and novelties in medicine, or about medical progress generally, they are apt to imagine that most other men think as they do; altogether overlooking the large proportion of practitioners who never cared about such things, and the equally large proportion who cared about them when young and comparatively idle, but whose attention the progress of years and active professional occupation have turned entirely into other channels. If the inquiry were made, I have little doubt that it would be found to be a fact, that a considerable proportion of the most eminent and best employed physicians and surgeons in the kingdom rarely buy any medical books, and still more rarely any journals. If they see the latter at all, they are contented with an occasional glance of them in their public or private clubs and societies.

It forms no part of my present business to explain or account for this singular disregard of medical literature by those best qualified to appreciate it and to promote it; but it is a circumstance, as already hinted at, not a little discreditable to the profession in this country. Yet who shall throw the first stone?

4. To the class of persons just mentioned, the expense of books is a matter of no moment, and is probably seldom considered by them; but it is certain that of the total number of practitioners in this country, there is a large proportion to whom the annual cost of a journal is a matter of consideration, sufficient, certainly, to preclude all idea of purchasing a second, if the expense of one has been already incurred.

The preceding explanations are applicable to journals generally: the following have more especial reference to the 'British and Foreign Medical Review.'

5. At the time the 'British and Foreign Medical Review' was established, in England the field was occupied by a Quarterly Journal having a large circulation. The great majority of the subscribers to this journal would naturally continue to take it; only a small proportion would take both journals, or would replace the old by the new. Even if the new journal were considered superior to the other, the substitution of the one for the other would not be a quick process. It is, nevertheless, well known that the establishment of the 'British and Foreign Medical Review' had a very unfavorable influence on the sale of its rival; as this sale certainly decreased after the appearance of the new journal, and fell at length to an amount considerably below that of its opponent. The existence of the two journals was thus mutually injurious in point of circulation: the competition, however, was doubtless valuable in other respects.

6. The very excellencies of the 'British and Foreign Medical Review,'—those high qualities which obtained for it the suffrages of the best judges,—were direct obstacles to its success with a large portion of the medical profession. It was pitched in too high a strain for the less educated practitioners. Its physiological and philosophical discussions, its investigation of principles, its generalizations, its correct but abstract and philosophical style, were, in a great measure, thrown away on this class of readers; they desiderated something lower, humbler, simpler; something they could at once grasp and understand. The mere "practical men" also,—men who look upon medical literature as a sort of manufactory, storehouse, shop, or waggon, for investigating, detecting, promulgating, and conveying "improvements in practice," by which they mean the announcement of a new remedy, or a modification of an old one, or its new or improved application in some unusual case, &c. &c.; in a word, some fresh addition to the already boundless stock of empirical conventionalities,—were sadly disappointed at finding in its pages general principles taking the place of individual cases, and therapeutical doctrines giving the go-by to bran-new specifics and charming formulæ without a chemical or pharmaceutical defect. I was early warned by many kind friends of the necessity of consulting the tastes of the classes just referred to; but I preferred to adhere to the fundamental principles of the Journal, knowing that these were in reality the *only true practical* principles, and hoping that the progress of years might bring greater intellectual capacity and more general enlightenment; or even that the Review might itself cure the evil from which it was meanwhile a sufferer. It has always been held expedient by the best judges to pitch the general tone of instruction rather above than below the capacity of the learners in any department of science, and I felt it my duty to adopt this rule. At the present moment I am far from regretting that such was my determination: I am sure the general result on the professional mind has been most beneficial: many individual instances of such benefit have come under my own observation.

7. Those who are accustomed to observe in ordinary life the wonderfully conciliating effect of praise and flattery, will be prepared to find these still more active in the world of letters, where, as is well known, vanity and the desire of literary and professional reputation are moving powers of the first order. It is accordingly familiar to the initiated how much all authors, but more especially those of the mediocre sort, are influenced by criticism of their writings. By weak men of this class, every commendatory or eulogistic notice of their works is set down as evidence of the critic's judgment, while every unfavorable report is attributed to ignorance, prejudice, or to some malignant motive. Now, when it is considered how very considerable a proportion of the medical profession in this country come forward in the present day, either as the writers of distinct works, or as the authors of communications in the numerous journals, it will be seen that the generally favorable or unfavorable *animus* of this class towards a journal, might thus have a considerable influence on its circulation. A man who is just hesitating whether he shall take-in a certain journal or not, may very likely be influenced *pro* or *con* by a favorable or unfavorable notice of his book or paper at the time; and a certain portion of the more sensitive will even be led by a severe though just criticism, to throw up the journal previously subscribed for. This is a fact well known to publishers. So well was this principle of action known to an editor whom I once knew, and so fully was its importance appreciated by him, that he adopted the system of general laudation as a commercial principle, and with great success. Now, in looking for the causes of the comparatively small sale of the 'British and Foreign Medical Review,' it is probably fair to reckon what has been called its *severity*, but which I call its impartiality and justice, as one of them. It is undoubtedly true that in its pages, quacks, cheats, pretenders of all sorts, and ignorant and illiterate writers of all ranks found little or no mercy; while even men of eminence were often severely criticised, without reference to their position in letters or station in society, but merely in relation to the actual merits or demerits of their books. It is equally true that never were there lavished in any journal more cordial and sincere commendations than were bestowed on works of real merit; but even in these cases, fair criticism, or even partial blame, was never withheld when it was thought due. Some instances of the curious effect of such mixed or neutralizing criticism on the minds of authors have come to my knowledge. And more than once, in the course of the publication of the Review, I have had an amusing illustration of the different way in which the same mind will view a criticism when directed at the production of another and at its own. I refer to the case in which a contributor has himself afterwards published a book. When this book has been reviewed by one of his colleagues, and undergone gentle criticism—it might possibly be somewhat more gentle on account of the previous relationship of the author to the Review—it has been amusing to see the quondam contributor exhibit the same sensitiveness to reproof as if he had never bestowed any on others; entirely forgetting how he himself had formerly laughed at (in others) the very feelings he now indulged as legitimate!

8. It appears from the Table that there has been a slight falling off in the sale during the last and present year. The greater part of this is easily explained by the fact that last year the Review began to be reprinted in America; consequently there has been a considerable decrease in the number of copies sent out to the United States. I think it likely, also, that a portion of the defalcation may be caused by the cry raised by some of the weekly Journals—pretty loud for a month or two—against certain articles in the Review on the subject of Homœopathy and Hydropathy.* These articles, while especially suited to the taste and judgment of the more philosophical members of the profession,—by whom, indeed, they were generally commended,—were likely enough to be misunderstood by the ill-informed generally, and especially by the numerous class of routineers whose knowledge of their profession is mainly empirical, and whose ideas of practice are almost entirely limited to the conventional administration of drugs. In no instance since the publication of the Review have I had more cause to be satisfied with the effect produced by any articles, than by these. In no case have I received such numerous, strong,

* The same cry will probably be raised against the excellent article on *Teetotalism* in the present number.

and cordial acknowledgments of the services rendered to medicine by the Review, as I have received and continue to receive on account of those articles, from all countries, and from the best men in them. I believe, indeed, that they have been the means of exciting a spirit of philosophical inquiry into the present state of therapeutics, which cannot fail to end in consequences most beneficial to medical science. It was amusing to observe, that while the weekly journals of general medicine were attacking these papers as *favoring* homœopathy (of course the writers of the attacks had never redd them), the Journals of Homœopathy were universally denouncing them as opposing it, and the great champions of the system of infinitesimals honoured them with numerous replies, and so-called refutations in the shape of pamphlets, letters, &c.

The various causes just enumerated seem to me satisfactorily to explain the comparatively limited sale of the Review, and its failure as a commercial speculation. Of course, there still remains *another* view of the matter, which those who choose may adopt for explaining the fact I have attempted to explain in my own way. It may be said that the Review failed because it *deserved* to fail—because, instead of being, as pretended, a very good commercial article, it was a very bad commercial article, and consequently could not and did not succeed commercially. As I have already committed myself on this question, I cannot here re-argue it. I leave it with confidence in the hands of honest men and competent judges.

As one of my principal objects in making the present exposition, is to aid future journalists in their attempts to serve the profession, I think it right to make one more observation on the financial part of the subject, before concluding. It must not be supposed from anything I have said, or from the inspection of the abstract of my accounts given below, that the financial arrangements of the Journal were heedlessly made or carelessly superintended. This was not the case. I believe that the sums paid to the printer, the paper-maker, and the publisher were really as moderate as they well could be, in fairness between man and man, regard being had to the quality of the materials and the work. The only thing in which a saving in these items might have been occasionally made, was *the size* of the different numbers. Eighteen sheets, or 288 pages, was the covenanted size of each number. It frequently happened, however, that when there was a pressure of what seemed interesting or important matter, the fixed limits were transgressed by the addition of one, one and a half, or even two sheets.* There was certainly no absolute necessity for this increase of bulk; and I dare say a rigid adherence to the original commercial covenant might have saved the proprietor about £40 or £50 annually. This is a point worth consideration for future proprietors and editors.

On the remuneration to contributors also, no doubt a saving *might* have been made. Small as my rate of payment was, compared with that received by contributors to respectable journals of general literature, still it was much beyond what had been customary in medical literature; and I dare say that if, on finding the Journal unprofitable, I had made the proposition to my literary friends to accept less for their labours, they would have readily agreed to it, as I am proud to say I always found them, to a man, influenced in their relations to the Journal by much higher considerations than those

* The average excess on the whole work was about one sheet and a half per volume, including the tables of contents, title-pages, and indexes. The present number contains twenty sheets.

of a pecuniary kind. By reducing the *honorarium* from six to five pounds per sheet, I might thus have saved from £60 to £70 per annum, which added to the saving just noticed, as practicable in paper and print, would have benefited the exchequer of the proprietary a good deal. I confess, however, that it never occurred to me to make this reduction; and I should be grieved to think that the future state of the medical press should render such a reduction necessary. Assuredly, the honorarium allowed by me was quite as small as accomplished scholars and experienced writers, like my literary friends, ought to accept; and was very inadequate remuneration for the time, and pains, and thought bestowed by many of them on their compositions. Still it is clear from the financial exposition now made, that one of two things must happen in future: either the profession in this country must be contented with an article produced at less cost,—probably, therefore, an inferior article,—or they must determine to become more extensively *purchasers* as well as *readers* of journals, if they will have them *good*, that is, produced at a certain expenditure.*

Although, in the preceding pages, I have had occasion more than once to

* A simple and ready way of augmenting the sale of journals, with little individual sacrifice, would be for each medical society or book-club to take two copies instead of one, of *good* journals. I know, from experience, how very *convenient* such an arrangement would be to the members of the societies generally; and its advantages in the way just hinted at are so obvious, that I cannot but hope that the hint may be taken. I would not have the rule made universal in regard to journals, but only applicable as to those which the members might deem deserving the distinction. Surely many large societies and large book-clubs might take, with great advantage to their members, not merely two, but three or four copies of some journals. The difference thus produced in the sale of a journal (probably 400 or 500 copies) might make all the difference between a journal struggling for existence and a journal highly prosperous as a commercial undertaking.

### *Account of the* Expenditure *and* Receipts *of th*[illegible]

EXPENDITURE.

| | 1836 | | | 1837 | | | 1838 | | | 1839 | | | 1840 | | |
|---|---|---|---|---|---|---|---|---|---|---|---|---|---|---|---|
| | £ | *s.* | *d.* | £ | *s.* | *d.* | £ | *s.* | *d.* | £ | *s.* | *d.* | £ | *s.* | *d.* |
| Paper, Printing, Engraving, Stitching . . . | 1166 | 1 | 1 | 745 | 10 | 1 | 659 | 0 | 10 | 666 | 4 | 11 | 742 | 9 | [illegible] |
| Advertising . . . | 49 | 17 | 6½ | 18 | 18 | 0 | 63 | 16 | 3 | 95 | 17 | 6 | 39 | 15 | [illegible] |
| Books for Review (purchased) | 71 | 11 | 4 | 18 | 7 | 8 | 48 | 16 | 0 | 44 | 0 | 2 | 49 | 7 | [illegible] |
| Contributors . . | 460 | 13 | 6 | 316 | 18 | 6 | 362 | 10 | 0 | 379 | 7 | 6 | 389 | 2 | [illegible] |
| Miscellaneous, including Carriage, Postage, &c. | 125 | 12 | 0 | 108 | 0 | 5 | 48 | 10 | 6½ | 70 | 17 | 6 | 79 | 4 | [illegible] |
| Total Expenditure . | 1873 | 15 | 5½ | 1207 | 14 | 8 | 1182 | 13 | 7½ | 1256 | 7 | 7 | 1299 | 19 | [illegible] |

RECEIPTS.

| | 1836 | | | 1837 | | | 1838 | | | 1839 | | | 1840 | | |
|---|---|---|---|---|---|---|---|---|---|---|---|---|---|---|---|
| For Books sold . . | 812 | 9 | 4 | 817 | 17 | 6 | 984 | 0 | 4 | 1161 | 12 | 4 | 1187 | 6 | [illegible] |
| Advertisements and Bills | 121 | 6 | 0 | 104 | 8 | 6 | 163 | 18 | 6 | 130 | 15 | 0 | 139 | 12 | [illegible] |
| Total Receipts . | 933 | 15 | 4 | 922 | 6 | 0 | 1147 | 18 | 10 | 1292 | 7 | 4 | 1326 | 18 | [illegible] |
| *Total number of copies sold each year, *including back numbers* . . | 3900 | | | 3985 | | | 4598 | | | 5573 | | | 5663 | | |
| *Average number of copies sold quarterly each year, *including back numbers* | 975 | | | 996 | | | 1149 | | | 1393 | | | 1415 | | |

* The numbers in these two lines show the total actual sale of the Journal for each year, and the total average sale f[illegible] each quarter; but they do not, by any means, give a true representation of the actual and relative sale of the *curre*[illegible] *numbers*, or what is usually termed the *circulation* of the Journal. Generally speaking, the numbers there given a[illegible]

refer to the pleasing relations existing between the Editor and the Contributors to the Journal, I should ill satisfy my feelings if I took leave of them without some more distinct acknowledgment of the claims they possess to my gratitude and sincere regard. It was no common fortune to occupy, for a series of years, a position which necessarily placed me in constant and close literary and personal intercourse with many of the most learned and intellectual members of the profession; which made me, for the time being, the prompter and, to a certain extent, the director of their studies, —the witness and, in a small degree, the sharer of their labours; and in which it was my especial function to communicate the fruits of these studies and labours to the medical world at large. It would be strange if the privileges and advantages of such a position could be even partially resigned without some pain and some regret. The parting with my late associates in their capacity of contributors, however, would be very different from what it now is, if I thought I was at the same time taking leave of them as friends and friendly correspondents. But I am thankful to say this is not so. It is most gratifying to me to believe that the breaking of the bond which constituted our formal literary union, leaves still untouched those nearer and dearer ties which gave to our official intercourse so cordial a character, and made the years devoted to it among the happiest as well as the most useful of my life:

> "nec me meminisse pigebit——
> Dum memor ipse mei, dum spiritus hos reget artus."

JOHN FORBES.

Old Burlington Street;
30*th September*, 1847.

British and Foreign Medical Review *from the Commencement.*

EXPENDITURE.

| 1841 | | | 1842 | | | 1843 | | | 1844 | | | 1845 | | | 1846 | | | 1847 | | | Total. | | |
|---|---|---|---|---|---|---|---|---|---|---|---|---|---|---|---|---|---|---|---|---|---|---|---|
| £ | *s.* | *d.* | £ | *s.* | *d.* | £ | *s.* | *d.* | £ | *s.* | *d.* | £ | *s.* | *d.* | £ | *s.* | *d.* | £ | *s.* | *d.* | £ | *s.* | *d.* |
| 604 | 1 | 7 | 560 | 5 | 6 | 622 | 7 | 3 | 516 | 8 | 6 | 594 | 17 | 2 | 580 | 2 | 0 | 567 | 18 | 9 | 8025 | 6 | 11 |
| 37 | 1 | 1 | 25 | 7 | 0 | 23 | 14 | 0 | 19 | 6 | 6 | 75 | 2 | 6 | 28 | 9 | 0 | 29 | 0 | 6 | 506 | 5 | 4½ |
| 33 | 4 | 5½ | 42 | 1 | 3 | 38 | 14 | 11 | 27 | 16 | 6 | 14 | 10 | 0 | 25 | 1 | 5 | 10 | 6 | 10 | 423 | 18 | 4½ |
| 367 | 7 | 6 | 387 | 10 | 6 | 380 | 18 | 6 | 424 | 14 | 0 | 427 | 10 | 6 | 369 | 19 | 0 | 401 | 12 | 0 | 4668 | 4 | 0 |
| 9 | 4 | 9 | 2 | 7 | 0 | 2 | 17 | 3 | 1 | 5 | 0 | 1 | 4 | 0 | 1 | 3 | 0 | 1 | 10 | 0 | 451 | 16 | 0½ |
| 1050 | 19 | 4½ | 1017 | 11 | 3 | 1068 | 11 | 11 | 989 | 10 | 6 | 1113 | 4 | 2 | 1004 | 14 | 5 | 1010 | 8 | 1 | 14075 | 10 | 8½ |

RECEIPTS.

| | | | | | | | | | | | | | | | | | | | | | | | |
|---|---|---|---|---|---|---|---|---|---|---|---|---|---|---|---|---|---|---|---|---|---|---|---|
| 1184 | 11 | 3 | 1057 | 0 | 7 | 1039 | 3 | 2 | 1067 | 8 | 1 | 1014 | 6 | 8 | 943 | 10 | 10 | 866 | 3 | 9 | 12135 | 10 | 8 |
| 134 | 2 | 0 | 100 | 0 | 0 | 100 | 0 | 0 | 100 | 0 | 0 | 100 | 0 | 0 | 100 | 0 | 0 | 100 | 0 | 0 | 1394 | 2 | 0 |
| 1318 | 13 | 3 | 1157 | 0 | 7 | 1139 | 3 | 2 | 1167 | 8 | 1 | 1114 | 6 | 8 | 1043 | 10 | 10 | 966 | 3 | 9 | 13529 | 12 | 8 |
| 5643 | | | 5451 | | | 5285 | | | 5481 | | | 5107 | | | 4919 | | | 4451 | | | | | |
| 1410 | | | 1363 | | | 1321 | | | 1370 | | | 1276 | | | 1229 | | | 1112 | | | | | |

greater than the current sale; and they are irregularly so, the amount of *back* numbers sold being much greater in some years than others. The sale of back numbers has considerably decreased during the last few years, which makes the diminution of the *circulation* during these years, appear much greater than it really has been.

P.S. Although the 'British and Foreign Medical Review,' as above stated, literally and absolutely closes its career with the present Number, I feel great satisfaction in being able to announce that it will be immediately succeeded by another Journal, which, I am given to understand, will be conducted on nearly the same plan and principles. The new Journal will have one great commercial advantage over the old, in having no English rival for the public favour, as it is arranged that there shall be an amalgamation of the two London Quarterly Journals into one, under the title of the 'British and Foreign Medico-Chirurgical Review.' And I am informed by my late excellent publisher,* the joint proprietor of the new Journal, that the same independent and liberal course, both as to criticism and literary remuneration, will be pursued as in the case of the Journal he so long published on my account. I have also reason to believe that many of the best writers in my Journal will be contributors to that which is to take its place. Although I shall not cease to feel great interest in the character and position of the new Journal, I think it right to state here, most explicitly, that I have and shall have no connexion whatever with it, as regards its proprietorship, composition, editing, or publishing, or in any other way, directly or indirectly. I sincerely hope for the honour and welfare of the profession, that it will be at least a GOOD JOURNAL; and I can conscientiously add, that if it shall prove to be a BETTER JOURNAL than that which I was able to supply (and with the experience of its predecessors to guide it, showing it as well what to imitate and what to eschew, it may reasonably be expected to be better), there will not be one among its readers who will more cordially rejoice than myself.

I hope my successor in the editorial chair, whoever he may be, will pardon me if I venture to offer him, as a parting legacy, a little advice for his guidance in his new and "Great Place." Although not so intended originally, these precepts are, like all that fell from the pen of the wise man who wrote them, of such universal application as to seem to be addressed specially to an Editor assuming office. At any rate, I am sure the Editor of the new Journal cannot but profit by giving heed to them.

"In the discharge of THE PLACE, set before thee the best examples; for Imitation is a globe of precepts. And after a time set before thee thine own example, and examine thyself strictly whether thou didst not best at first. Neglect not also the examples of those that have carried themselves ill in the same place; not to set off thyself by taxing their memory, but to direct thyself what to avoid. Reform, therefore, without bravery or scandal of former times and persons; but yet set it down to thyself, as well to create good precedents as to follow them. Reduce things to the first institution, and observe wherein and how they have degenerated; but yet ask counsel of both Times, of the ancienter Time what is best, and of the latter Time what is fittest. Seek to make thy course regular, that men may know beforehand what they may expect: but be not too positive and peremptory; and express thyself well when thou digressest from thy rule. Preserve the Right of thy Place, but stir not questions of jurisdiction; and rather assume thy right in silence and *de facto*, than voice it with claims and challenges. Preserve likewise the rights of Inferior Places; and think it more honour to direct in chief than to be busie in all. Embrace and invite helps and advices touching the execution of thy Place; and do not drive away such as bring thee information, as meddlers, but accept of them in good part. . . . . . . . . Use the memory of thy Predecessor fairly and tenderly; for if thou dost not, it is a debt will sure be paid when thou art gone. If thou have Colleagues respect them, and rather call them when they look not for it than exclude them when they have reason to be looked to be called."
—Bacon, *Of Great Place.*

J. F.

* I cannot let this last opportunity pass without expressing my sincere thanks to Mr. Churchill for his uniform liberality and kindness, and his great attention to the affairs of the Journal.

### INDEX TO VOL. XXIV

OF THE

### BRITISH AND FOREIGN MEDICAL REVIEW.

---

PAGE

Acton, Mr. case of monstrosity . 325
Age, influence of, on health and mortality . . . . . 111
Albumen, fluid, Dr. Von Bibra on . 287
Albuminuria, iodide of iron in . 44
analysis of the blood in 45
Alcoholic drinks, physiological effects of 515
Dr. Dalton Hooper on 532-4
Alderson, Dr. on diseases of the stomach 273
Analogy of the brain, spinal cord, and sympathetic ganglia . 203
between instinctive, reflex, and emotional actions . 207
Anemia, treatment of . . 44
Aneurism, peculiar case of . . 323
Mr. Crisp on . . 414
internal . . 416
thoracic . . . ib.
of abdominal aorta . 417
dissecting . . . 418
cerebral . . ib.
external . . . 419
of the innominata . . 420
of the subclavian . ib.
circoid . . . 421
of iliac arteries . . ib.
popliteal . . . ib.
carotid . . . . ib.
Aneurisms, false, Mr. Liston on . 326
Angina pectoris, nitrate of silver in 41
tonsillaris, treatment of . 138
Animal physiology, Dr. Unzer on 181
bodies, mechanical machines of 194
Animals, domestic, various diseases of the . . . . 83
Ansted, Prof. the ancient world . 281
Aphthæ, habitat of . . . 425
microscopical character of ib.
description of . . 427
colour of . . 428
causes of . . . 430
Arnott, Mr. on wound of tibial artery 323

PAGE

Arteries and veins, structure of . 410
Mr. Crisp on diseases of the ib.
morbid conditions of the . 411
of the extremities, inflammation of the . . 412
Arteritis . . . 411
Artificial pupil, treatise on . . 364
Atmosphere, influence of, on health 98
Atrophy of the face . . 43
Baly, Dr. on dysentery . . 333
Barkow, Dr. on hybernation . 408
Baumgärtner, Dr., his physiology and practical medicine . . 129
Bellini on metastasis . . . 288
Berg, Dr. on thrush in children . 422
Bibra, Dr. Von, on zoochemical substances . . . . . 286
Bini, Dr., clinical medicine . . 360
Bone, on the formation of . . 386
Booth, Rev. J. on education . . 284
Botany, Mr. Henfrey on . . 223
Brain, diseases of . . . 89
physiology of the . . 185
disease of . . . 323
and spinal marrow . 324
human, Mr. Solly on . . 572
Brett, Dr. on cataract, &c. . 364
his plagiarisms from Dr. Mackenzie . . 365
Bright's disease . . . 521
British and Foreign Medical Review, postscript to 585
expenditure and income of 596
Bufalini, Prof. clinical medicine . 360
Busk, Mr. on abscess . . 331
Cancer, Dr. Hannover on . . 373
Dr. Hannover's definition of 374
Cancer-cells, description of . . ib.
Casper, Dr. on medical statistics . 91
Cataract, &c., Dr. Brett's treatise on 364
Cells, Baumgärtner's claims to discovery of . . . 135

PAGE
Chambers, Mr. Robert, his memoir of Dr. A. Combe . . 581
Charlton, Dr. on scarlatina . . 285
Children, thrush in . . 422
Chloride of sodium, importance of, in the formation of blood . . 270
Cholera, Asiatic . . . 146
Chorea, treatment of . . 41
Combe, Dr. Andrew, memoir of . 581
Conolly, Dr. on lunatic asylums . 156
Constitution and predisposition . 303
Copland, Dr. on dysentery . . 333
Crisp, Mr. on diseases of blood-vessels 410
Cumming, Mr. on the eye . . 330
Death . . . . 320
Desire and aversion, nature of 191, 198
Diabetes mellitis, condition of the kidneys in . . . . 45
Diaphragm, on diseases of . . 166
anomalous motions of the 168
wounds of the . . 170
rupture of the . . 172
spasmodic action of the . 173
tremulous motion of the . ib.
convulsive action of the . ib.
clonic spasm of the . 174
tonic spasm of the . . 175
paralysis of the . . 176
rheumatism of the . . 177
inflammation of the . 178
pleurisy of the . . ib.
malformations of the . ib.
hernia of the . . ib.
adhesions of the . . 180
parasitic formations on the ib.
Diarrhœa of infants, nitrate of silver in 47
Digestive apparatus, diseases of . 82
organs, inflammatory products of 124
Disease, idea of . . . 297
nature of . . . ib.
disposition to . . 301
hereditability of . . 303
symptoms of . . 304
course of . . . 315
events of . . . 318
Diseases, cachectic . . 87
inflammatory . . 110
severe, Dr. Seymour on . 139
of the stomach . . 273
local and general . 305
Dixon, Mr. on tumour . . 327
Dowler, Dr. experimental researches 279
Dunlop, Mr. John, his exertions in the cause of temperance . 521
Dysentery, treatises on . . 333
simple, non-contagious . 334
complicated . . 345
Dyspepsia, Dr. Steward on . 579
Ear, statistics of diseases of . . 22
treatment of various diseases of 37

PAGE
Ear-medicine, contribution to . 22
Ectopia . . . . 179
Education, medical, Mr. Green on . 216
Rev. J. Booth on . 284
Emotions and passions . . 200
Empyema, treatment of . . 47
Epilepsy, phenomena of . . 42
Etiology, general . . . 298
Eye, luminosity of the . . 330
Ferrario, Dr. on the mortality in the Milan Hospital . . . 379
Fever, nervous . . . . 110
in Ireland, Mr. Kennedy on . 285
Fibrils, two sets of, in each nerve . 193
Follicular disease, anatomy of . 492
causes of . 493
symptoms of . 495
treatment of 497-504
Flourens, M. on formation of bone . 386
Fluids, Vogel on . . . 276
Food, chemistry of . . . 264
Gangrene of the mouth . . 46
Gastric juice . . . 272
Gastritis, milk diet in . . 47
Ghinozzi, Dr. clinical medicine . 360
Giacomini, Prof. clinical medicine . ib.
Glaisher, Mr. hygrometrical tables . 283
Gout . . . . 147
Green, Dr. on diseases of the air-passages . . . . 482
Mr. mental dynamics . 216
Gunsburg, Dr. his pathological histology . . . . 118
Hannover, Dr. pathology of cancer . 373
Harty, Dr. on dysentery . . 333
Hemicrania, nitrate of silver in . 41
Hemorrhage, colourless . . 138
secondary . . 419
Henfrey, Mr. outlines of botany . 223
Henle, Prof. manual of rational pathology . . . . 289
Henoch, Dr. clinical observations . 41
Hernia, diaphragmatic . . 178
Heusinger, on comparative pathology 81
Hewett, Mr. on aneurism . . 323
Homœopathy, Dr. Sankey on . . 215
Hunterian oration, Mr. Green's . 216
Hunt, Mr. on diseases of the skin . 280
Hutchinson, Mr. on detecting disease by the spirometer . . 327
Hybernation, Dr, Barkow on . 408
Idiotcy, definition of . . . 2
psychological symptoms of . ib.
Idiots, on the education of . . 1, 3
muscular system in 4
touch in . . 6
taste and smell in . ib.
ear in . . 7
speech in . ib.
sight in . . 9

PAGE
Idiots, moral treatment of . . 14
Impressions, centric and peripheral 201
centric diffusion of . 207
Inflammatory products of various tissues . . . . . 121
Inosinic acid . . . 267
Insanity, importance of cleanliness in 159
open-air exercise in . 160
on diet and employment in 163
Dr. Mayo on . . . 225
Instinct, nature of . . . 192
Instincts, sensational . . . 199
Intemperance the great cause of crime 517
Intestines, diseases of . . . 83
Irritants, general action of . . 299
Johnson, Dr. on Bright's disease . 321
Kennedy, Mr. on famine and fever in Ireland . . . 285
Kidney, inflammatory products of the 126
structure and changes of the 331
Kramer, Dr. on diseases of the ear . 22
Kreatinine, preparation of . . 266
Lactic acid . . . 268, 278
Larynx, diseases of . . . . 482
Lee, Mr. on tumours of the uterus . 506
Liebig, chemistry of food . . 264
Liston, Mr. on wounded arteries . 326
Liver and bile, diseases of . . 84
inflammatory products of the . 125
Lonsdale, Mr. on lateral curvature of the spine . . . 578
Lunatic asylums, Dr. Conolly on . 156
attendants in . 163
religious services in 164
superintendents of ib.
Lungs, inflammatory products of . 123
Luxford, Mr. his phytologist . . 436
Lymphatic system, lesions of the . 84
M'Arthur, Dr. directions to seamen . 212
Material ideas, Unzer on . . 186
action of, on the brain 192
nerves 193
Mayo, Dr. on insanity . . 225
Medical statistics . . . 91
Medicines, list of, for ships . 213
Medico-Chirurgical Transactions, vol. xxix . . . . 321
Mehliss, Dr. on diseases of the diaphragm . . . . 166
Memoir of Dr. Andrew Combe . 581
Meningitis, infantile, treatment of . 41
Mental derangement . . 150
Metastasis, Bellini on . . . 288
Monstrosity, case of . . 325
Morehead, Dr. dysentery in Bombay 333
Morphology, a contribution to . 135
Mortality, various causes of . . 113
in the Milan hospital . 379
Motion, organic . . . 133

PAGE
Mouth, diseases of the . . 82
Mucous membranes, inflammatory products of . . . 120
Mugna, Dr. clinical medicine . . 360
Muscular fibre, structure of . 128
Natural history of man, Dr. Prichard on . . . . 49, 441
Nerves, structure of, &c. . . 131
biotic properties of the . 187
incident excitor and reflex-motor . . . 205
Nervous system, diseases of . 89
Newman, Mr. his zoologist . . 436
Nitrate of silver, its use in laryngeal diseases . . . . 497
Osseous system and joints, affections of 87
Paget, Mr. on malformation of the brain 323
Paraplegia, causes and treatment of 42
Parasitoid formations, nature of . 135
Passions and emotions . . 200
Pathology, comparative, Heusinger on 81
rational, Prof. Henle on . 289
Pathological histology . . 118
Pemphigus, chronic, case of . . 48
Pericarditis, Dr. Taylor on . 548
friction-sound of . . 549
heart-sounds in . 551
cardiac dullness in . 552
heart's impulse in . ib.
undulatory movement in ib.
tactile vibration in . 553
cardiac bulging in . ib.
failure of diagnosis in . 554
symptoms of . . 556
course of . . 559
use of mercury in . 565
causes of . . 566
Peritonitis in children, treatment of 47
Phthisis, table of mortality from . 106
Physical history of mankind . 49, 441
Phytologist, the . . . . 437
Plague, Dr. Prus on . . 242
Dr. White on . . ib.
Pleasure and pain, physiological nature and operation of . . 197
Pleurisy, diaphragmatic . . 178
Postscript to the Review . . 585
Prichard, Dr. natural history of man 49, 441
physical history of mankind . ib.
Proceedings of the World's Temperance Convention . . 515
Products, inflammatory . . 119
Psoriasis inveterata, treatment of . 48
Prus, Dr. on plague and quarantine 242
Pulsation, abdominal . . 413
Quain, Dr. elements of anatomy . 386
Quarantine, correspondence concerning 242
English regulations . 263
French regulations . ib.

PAGE
Rainey, Mr. on the arachnoid membrane 324
Redford, Mr. on the body and soul . 287
Reflex acts, Unzer on . . . 195
action, laws of . . . 201
centric . . 206
doctrines of, propounded by Unzer . . 210
phenomena, analysis of . 205
theory, Dr. Dowler on . 279
Respiratory organs, diseases of, in the lower animals . . 84
Rheumatism, acute, treatment of . 45
Romberg, Dr. clinical observations . 41
Sankey, Dr. instructions for merchant seamen . . . . . 212
Sarcosine . . . . . 267
Scarlatina, treatment of the sequelæ of 45
Dr. Charlton on . . 285
Sciatica, treatment of . . 154
Scirrhus of the œsophagus . . 46
Scott, Dr. on the education of idiots 1
Scrofula, prison, causes of . . 138
Seamen, Drs. Sankey and M'Arthur on 212
Season, influence of on health . . 92
Séguin, on the treatment and education of idiots . . . 1
Serous membranes, inflammatory products of . . . . . 119
Sensation, mode of . . 132
Sexual organs, affections of . . 87
Seymour, Dr. his thoughts . . 139
Sharpey, Dr. on bone . . 386
Skin, diseases of in various animals . 85
external, inflammatory products of 122
general structure of . . 130
diseases of . . . 280
Skull, human, form of the . . 381
Solly, Mr. on the human brain . 572
Spinal marrow, diseases of . . 89
Spine, lateral curvature of . . 578
Spirometer, Mr. Hutchinson on the . 327
Spitta, Mr. on cyanosis . . 324
Steward, Mr. on dyspepsia . . 579
Stomach, diseases of in lower animals 83
diseases of . . 141, 273
Strabismus, &c., treatise on . 364
Subclavian artery, ligature of . . 322
Sympathies, abnormal . . 314
normal . . . 307

PAGE
Sympathy and antagonism . . 306
Table of expenditure and income of the Review . . . 596
Taylor, Dr. John, on pericarditis . 548
Teetotalism . . . 515
Temperance and teetotalism . . ib.
Temperature, influence of on health 96
Thermometers, Mr. Glaisher on . 283
Thomson, Dr. on food . . 332
Throat, Dr. Green on diseases of . 482
Thrush, in children . . . 422
definition of . . 423
microscopical character of . ib.
a parasite . . . 426
a contagious disease . . 431
prevalence of . . 432
danger of . . . ib.
treatment of . . 433
Tissues, opposite polarity of the . 133
Tobacco, a cause of disease of the throat . . . . . 494, 506
Tomes, Mr. on osseous tissue . 386
Tonsils, follicular disease of the . 489
Toynbee, Mr. on the human kidney . 331
Tuberculosis, Dr. Gunsburg on . 127
Tumours of the uterus, Mr. Lee on . 506
symptoms of 507
treatment of . 509
Typhus fever, treatment of . . 137
Unzer, Dr. physiological inquiries . 181
principles of animal physiology ib.
biographical notice of . . 210
Urinary organs, disease of in lower animals . . . . . 86
Uterus, Mr. Lee on tumours of the . 506
Uvula, follicular disease of the . 490
Vascular system, disease of the . 84
Vincent, Mr. cases of disease of brain 323
Vogel, on the mixing of fluids . . 276
Voluntary muscles, instinct of motion of . . . . 199
Warren, Dr. on ligature of left subclavian . . . . . 322
Weather, influence of, on health and life 92
White, Dr. on plague and quarantine 242
Wright, Dr. introductory lecture . 282
Zenne, Dr. on the form of the human skull . . . . . 381
Zoologist, the . . . . 439

END OF VOL. XXIV.

C. AND J. ADLARD, PRINTERS, BARTHOLOMEW CLOSE.

THE BRITISH AND FOREIGN MEDICAL REVIEW.

NO. XLVIII.—OCTOBER, 1847.

### CONTENTS.

### Analytical and Critical Reviews.

PAGE

1. Professor Henle's Manual of Rational Pathology . . . 289
2. Medico-Chirurgical Transactions . . . . . . 321
3. Harty, Baly, Copland, and Morehead on the Pathology and Treatment of Dysentery . . . . . . . . 333
4. Bufalini and Giacomini's Clinical Medicine . . . . . 360
5. Dr. Brett on Cataract, Artificial Pupil, and Strabismus . . . 364
6. Dr. Hannover on the Pathology of Cancer . . . . 373
7. Dr. Ferrario on the Mortality of the Milan Hospital . . . 379
8. Dr. Zenne on the Form of the Human Skull in its Ethnographical Relations 381
9. Flourens, Sharpey, Tomes on the Formation of Bone . . . 386
10. Dr. Barkow on Hybernation . . . . . . 408
11. Mr. Crisp on the Diseases and Injuries of Arteries . . . 410
12. Dr. Berg on the Thrush in Children . . . . . 422
13 The Phytologist and Zoologist . . . . . 436
14. Dr. Prichard on the Physical and Natural History of Mankind . . 441
15. Dr. Green on Diseases of the Throat and Larynx . . . 482
16. Mr. Lee on Tumours of the Uterus and its Appendages . . . 506
17. The Physiological Effects of Alcoholic Drinks: Temperance and Teetotalism 515
18. Dr. Taylor on Pericarditis . . . . . . 548
19. Mr. Solly on the Human Brain . . . . . . 572
20. Mr. Lonsdale on Curvature of the Spine . . . . 578
21. Dr. Steward on Dyspepsia . . . . . . 579

The late Dr. Andrew Combe . . . . . . 581

Postscript to No. XLVIII of the British and Foreign Medical Review . 585

Index, Title, &c.

*INDEX to the British and Foreign Medical Review.*

A COMPLETE GENERAL INDEX TO THE WHOLE TWENTY-FOUR VOLUMES has been some time in preparation, and will be published in the course of a few months.

